# Effectiveness of Single Miniplate Fixation for Mandibular Symphysis and Parasymphysis Fractures: A Clinical Study

**DOI:** 10.7759/cureus.94946

**Published:** 2025-10-19

**Authors:** Pranjali P Kulkarni, Amod P Patankar, Swapna A Patankar, Sudhir Pawar, Kaustubh Kulkarni

**Affiliations:** 1 Oral and Maxillofacial Surgery, Bharati Vidyapeeth (Deemed to be University) Dental College and Hospital, Pune, Pune, IND; 2 Oral Pathology and Microbiology, Bharati Vidyapeeth (Deemed to be University) Dental College and Hospital, Pune, Pune, IND

**Keywords:** arch bar, cost-effective fixation, mandibular fracture, maxillofacial trauma, miniplate osteosynthesis, nerve injury, parasymphysis, symphysis, tension band

## Abstract

Introduction

Symphysis and parasymphysis fractures are common in maxillofacial trauma, requiring precise management to restore form, function, and aesthetics. While Champy’s technique recommends two miniplates - superior and inferior - for optimal stability, placing a superior plate in the canine-premolar region risks damaging dental roots and neurovascular structures. Additionally, the cost of dual plating may be a burden in low-resource settings. This study evaluates the efficacy of a single miniplate at the inferior border, aided by an arch bar as a tension band, for managing these fractures.

Methods

This prospective clinical study included 15 patients presenting with isolated mandibular symphysis or parasymphysis fractures. All fractures were managed using a single 2.5 mm miniplate along the inferior mandibular border, with an arch bar serving as a tension band across the dental arch. Patients were evaluated postoperatively for the incidence of surgical site infection, wound dehiscence, malunion or nonunion, malocclusion, miniplate fracture, and neurosensory deficits. Cost implications related to avoiding the use of a second miniplate were also analyzed. Follow-up was conducted for a minimum of four weeks postoperatively.

Results

The study observed that single miniplate fixation in combination with an arch bar provided satisfactory clinical outcomes in most cases. There were no significant incidences of postoperative infection, plate fracture, or malocclusion. Neurosensory complications were minimal and comparable to standard dual-plate protocols. Importantly, the reduction in hardware usage translated to lower overall treatment costs, offering a substantial benefit for patients from economically constrained backgrounds.

Conclusion

Single miniplate fixation along the inferior border of the mandible, when augmented with a tension band in the form of an arch bar, appears to be a reliable and cost-effective alternative for the management of symphysis and parasymphysis fractures. This technique minimizes iatrogenic risk to adjacent anatomical structures and offers a financially feasible solution, particularly in low-resource settings. Larger randomized controlled studies are recommended to further validate these findings and support broader adoption of this simplified approach in clinical practice.

## Introduction

Fractures of the mandibular symphysis and parasymphysis are among the most frequently reported injuries of the lower jaw. Although the mandible is the largest and most robust facial bone, its prominent anatomical position makes it particularly vulnerable to trauma. Specific anatomical regions of the mandible are more susceptible to fracture due to structural weaknesses, such as the cantilevered angle, narrowed condylar neck, presence of the mental foramen, and the elongated canine root [1].

The symphysis is one of the three most common sites of mandibular fracture, following the angle and condyle, and accounts for approximately 18-20% of cases in adults [2]. Based on the classification proposed by Dingman and Natvig, the symphysis lies between vertical lines distal to the canines, while the parasymphysis extends from the distal root of the lateral incisor to the distal root of the canine. Accurate anatomical classification is essential for appropriate diagnosis and surgical planning.

The biomechanics of anterior mandibular trauma are complex due to muscular influences, particularly from the masseter and temporalis muscles, which exert forces that displace the inferior border at the fracture site [3]. Common etiological factors include road traffic accidents, interpersonal violence, falls, sports-related injuries, occupational trauma, firearm injuries, and pathological conditions [4].

The primary goals in managing mandibular fractures are to restore pre-existing anatomical form, ensure functional occlusion, and preserve facial aesthetics. For fractures in the symphysis and parasymphysis regions, desired outcomes include elimination of pain, achievement of proper occlusion, restoration of a functional inter-incisal opening of approximately 40 mm, and re-establishment of mandibular and facial symmetry [5].

One of the most significant developments in fracture management has been the use of miniplate osteosynthesis. This approach is based on Champy’s concept of ideal osteosynthesis lines, which recommends placing fixation devices along the biomechanical zones of tension and compression [6]. For symphysis and parasymphysis fractures, which are subject to significant torsional and tensile forces, standard protocols advocate for the use of two miniplates - one at the superior (tension-bearing) border and another at the inferior (compression-bearing) border [7].

However, placement of a miniplate along the superior border in the canine-premolar region carries an increased risk of iatrogenic injury to the mental and inferior alveolar nerves, or damage to dental roots, due to limited anatomical space [8]. To mitigate these risks, clinicians may opt to place a single miniplate along the inferior border. This technique typically necessitates the addition of a tension band - most commonly in the form of an arch bar - to stabilize tensile forces at the superior margin. Moreover, in low-resource settings such as India, the additional cost of a second miniplate may be prohibitive, especially for patients from lower socioeconomic backgrounds [9].

This study aims to assess the clinical efficacy and necessity of using a second miniplate in the management of symphysis and parasymphysis fractures. It evaluates patient outcomes following fixation with a single miniplate placed along the inferior mandibular border, supported by an arch bar, by examining rates of soft or hard tissue infection, wound dehiscence, malunion, nonunion, malocclusion, hardware failure, treatment costs, and iatrogenic neurosensory complications.

## Materials and methods

Study design

This prospective clinical study was conducted from 01/09/23 to 10/01/25 in the Department of Oral and Maxillofacial Surgery at Bharati Vidyapeeth Dental College and Hospital, Pune. Fifteen patients with mandibular symphysis or parasymphysis fractures meeting the inclusion criteria were enrolled. Ethical approval was obtained prior to data collection by the institutional Ethics Committee (EC/NEW/IMST/2021/MH/0029). 

Sample size justification

The sample size was calculated using Scalex and ScalaR calculators [10]. Based on data from previous studies evaluating comparative postoperative outcomes following placement of a single miniplate with an Ehrich arch bar versus two miniplates for the fixation of symphysis and parasymphysis fractures, considering the expected prevalence to be 96%, the desired precision (corresponding to effect size) to be 10%, and the level of confidence at 95% (z=1.96), the sample size estimated was 15.

Statistical analysis

All surgical procedures followed standard aseptic protocols, and postoperative care included cold compress application and routine antibiotic and analgesic coverage. Data were compiled in Microsoft Excel (Redmond, WA, USA) and analyzed using SPSS version 23 (IBM Corp., Armonk, NY, USA), keeping the level of significance at 5%. Descriptive statistics were presented as mean ± standard deviation.

Inclusion criteria

Patients aged between 18 and 65 years who presented to the Department of Oral and Maxillofacial Surgery and who were indicated for open reduction and internal fixation (ORIF) with plating under general anesthesia (GA) for the treatment of simple or unfavorable (displaced and undisplaced) fractures in the mandibular symphysis/parasymphysis region were included in the study.

Exclusion criteria

Active infections, comminuted fractures, panfacial trauma, or patients with edentulous mandibles. Mentally challenged or non-communicative patients and patients unwilling or non-compliant with follow-up and American Society of Anesthesiologists (ASA) III or higher (i.e., medically unfit for GA) were excluded from the study.

Outcome variables

The study's primary outcome variable was to evaluate the following parameters: wound dehiscence and incidence of bone or soft tissue infection. Malocclusion, plate exposure, paresthesia in the mandibular or inferior alveolar nerve distribution, plate fracture, and tenderness and mobility at the fracture site following single plate fixation for treatment of symphysis and parasymphysis fractures.

Surgical protocol

**Figure 1:**
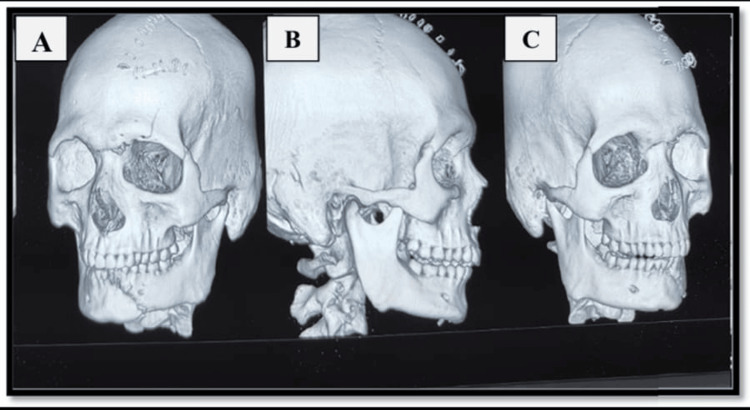
Preoperative three-dimensional (3D) CT of patient with displaced fracture of left parasymphysis of mandible: A) left oblique view; B) right view; C) right oblique view

**Figure 2:**
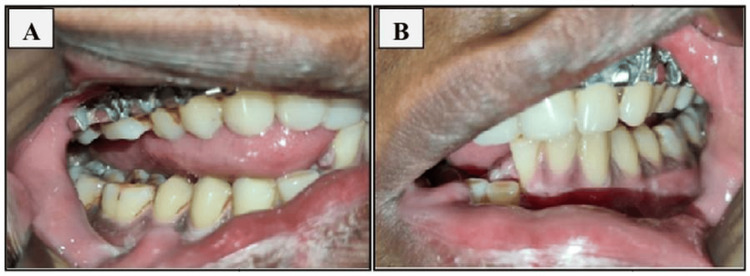
Preoperative occlusion of the patient; arch bar in situ with upper arch: A) right side; B) left side

Preoperative imaging of an orthopantogram or a three-dimensional conventional tomography scan was done (Figure 1). Preoperative occlusion of the patient was also noted (Figure 2). Antibiotics (amoxicillin-clavulanic acid 1.2 g) were administered intravenously one hour preoperatively. Intermaxillary fixation (IMF) using Erich arch bars and stainless steel wires was done.

**Figure 3:**
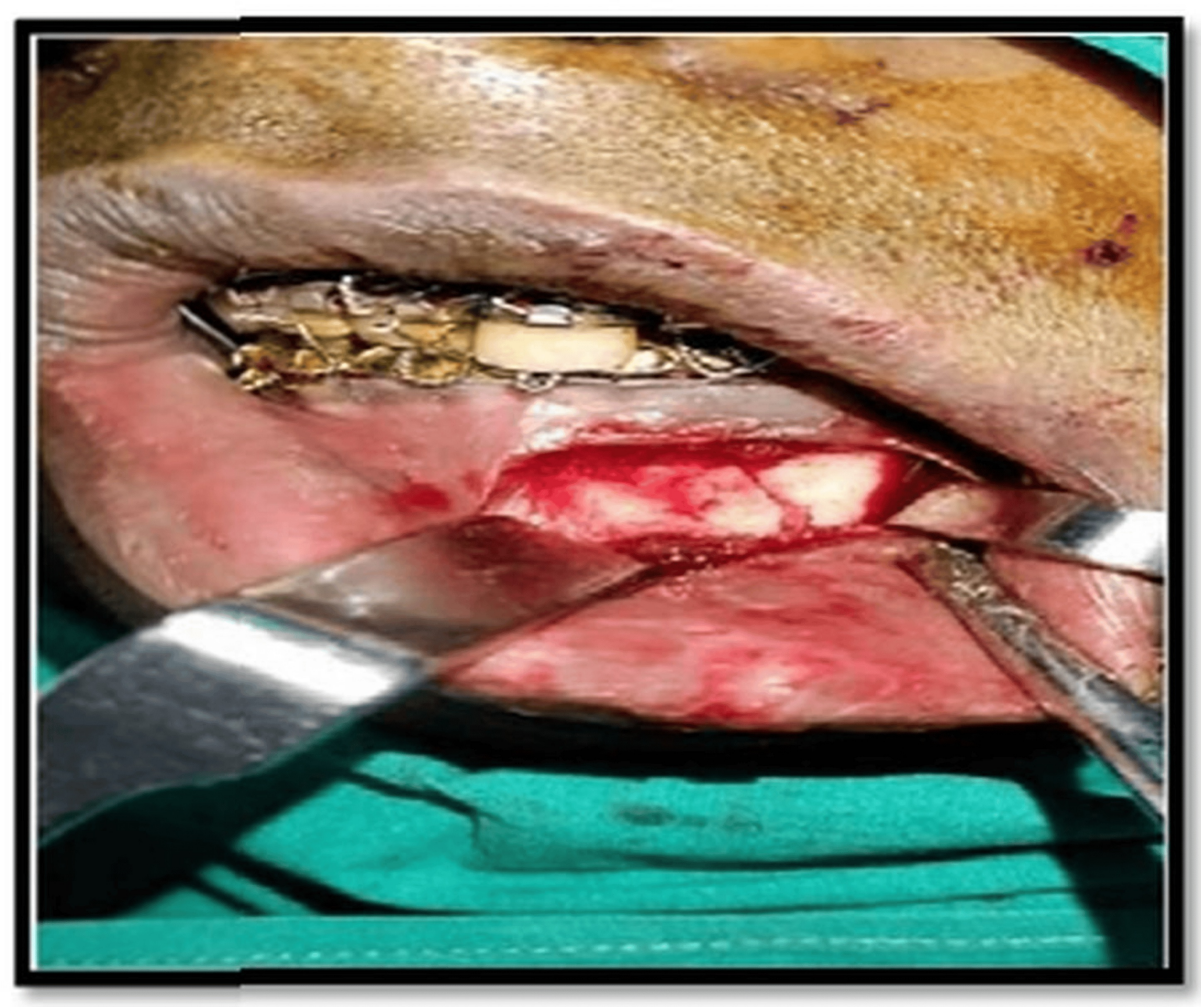
Intraoperative photo of the fracture site; intermaxillary fixation (IMF) done

Under stringent aseptic conditions and local anesthesia, submucosal infiltration using lignocaine with adrenaline (1:80,000) was used to treat all patients. A standard mandibular vestibular incision was placed intraorally, and a full-thickness mucoperiosteal flap was raised until the fracture of the symphysis or parasymphysis was exposed (Figure 3). 

**Figure 4:**
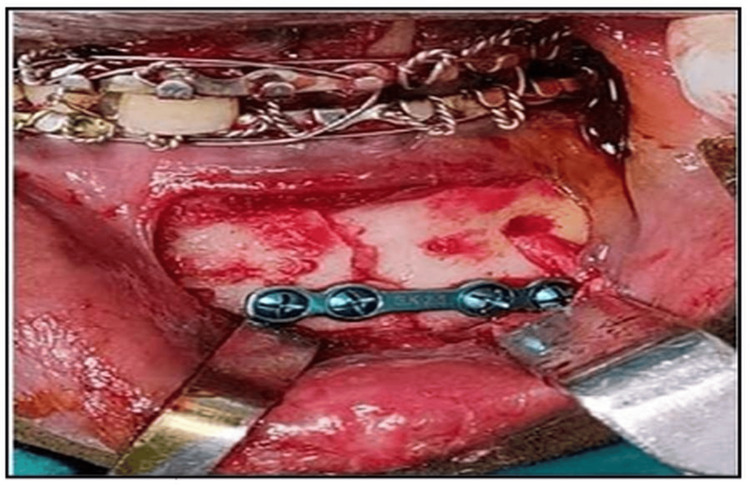
Fixation done with 2.5 mm titanium plate (four-hole with gap) and four 2.5 mm X 10 mm screws

**Figure 5:**
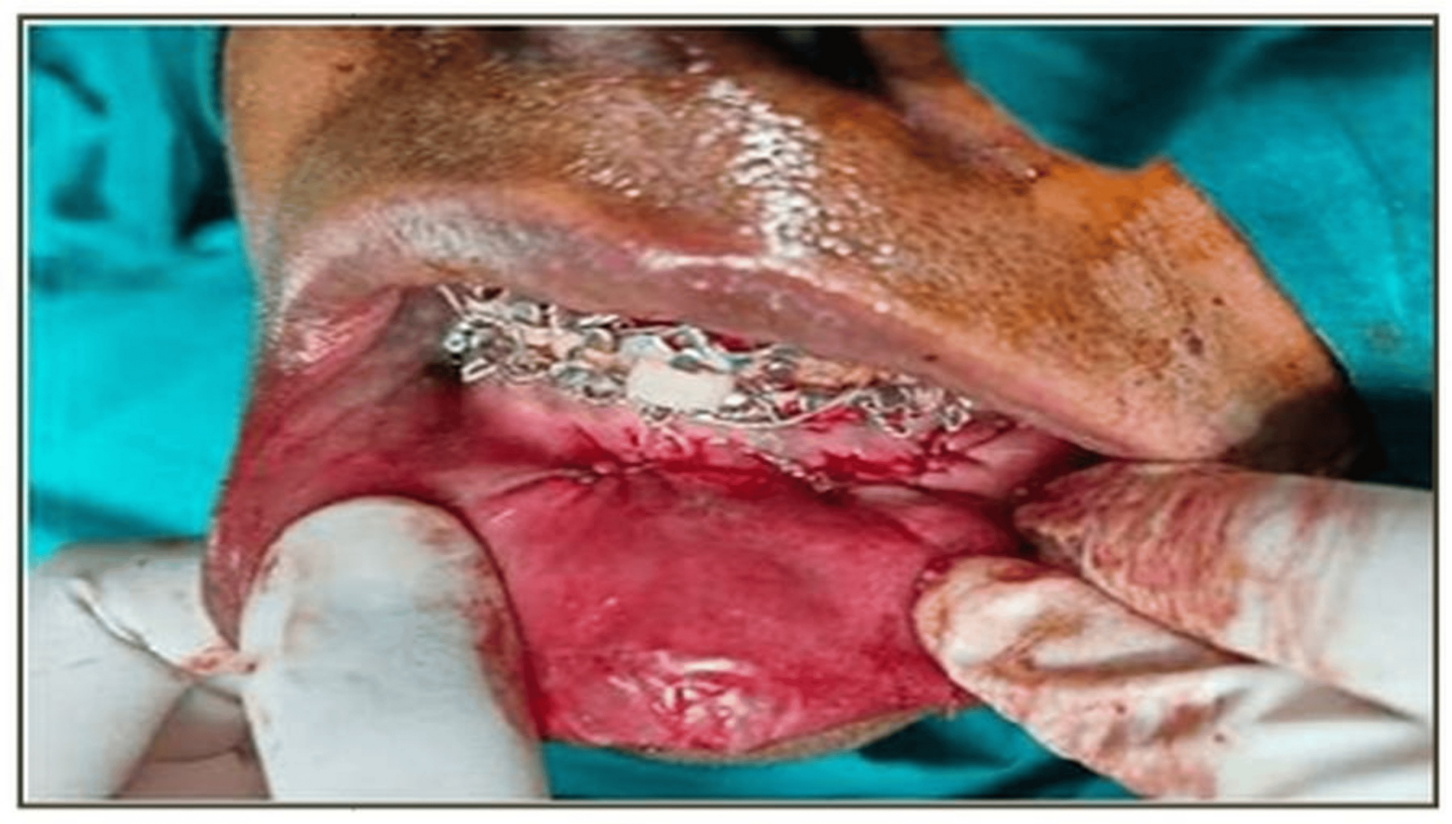
Primary closure done with Vicyrl 3-0

Once the surgical site was exposed, the fracture segments were carefully elevated or reduced. Fixation was done with a single 2.5 mm titanium miniplate (four-hole with gap) using 2.5×10 mm screws (Figure 4). The IMF was released after fixation and primary closure was done with Vicryl 3-0 suture (Figure 5). The arch bar was retained for four weeks postoperatively, and the patients were instructed on oral hygiene.

Postoperative assessment and follow-up

**Figure 6:**
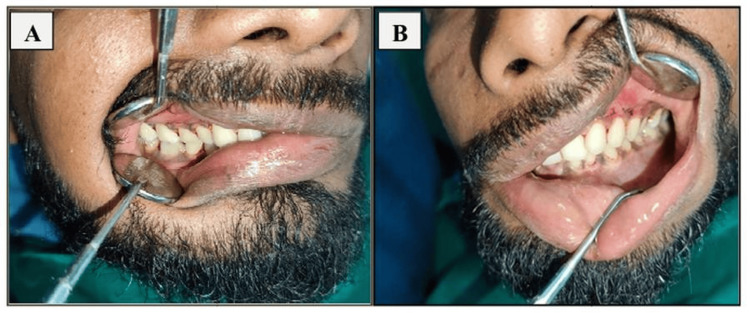
Postoperative occlusion at the end of four weeks; post arch bar removal: A) right side; B) left side

**Figure 7:**
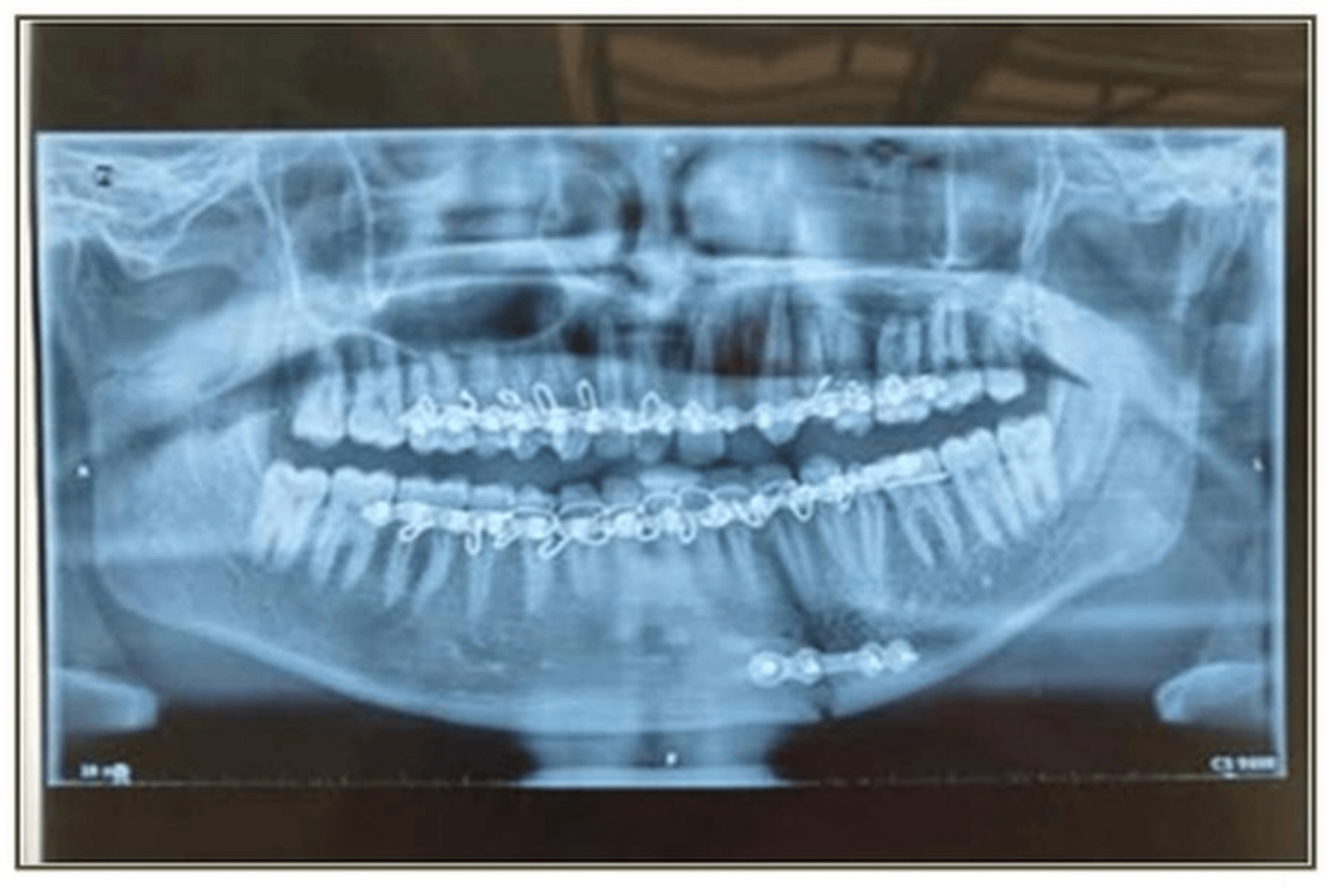
Postoperative orthopantomogram (OPG) at the end of one week

**Figure 8:**
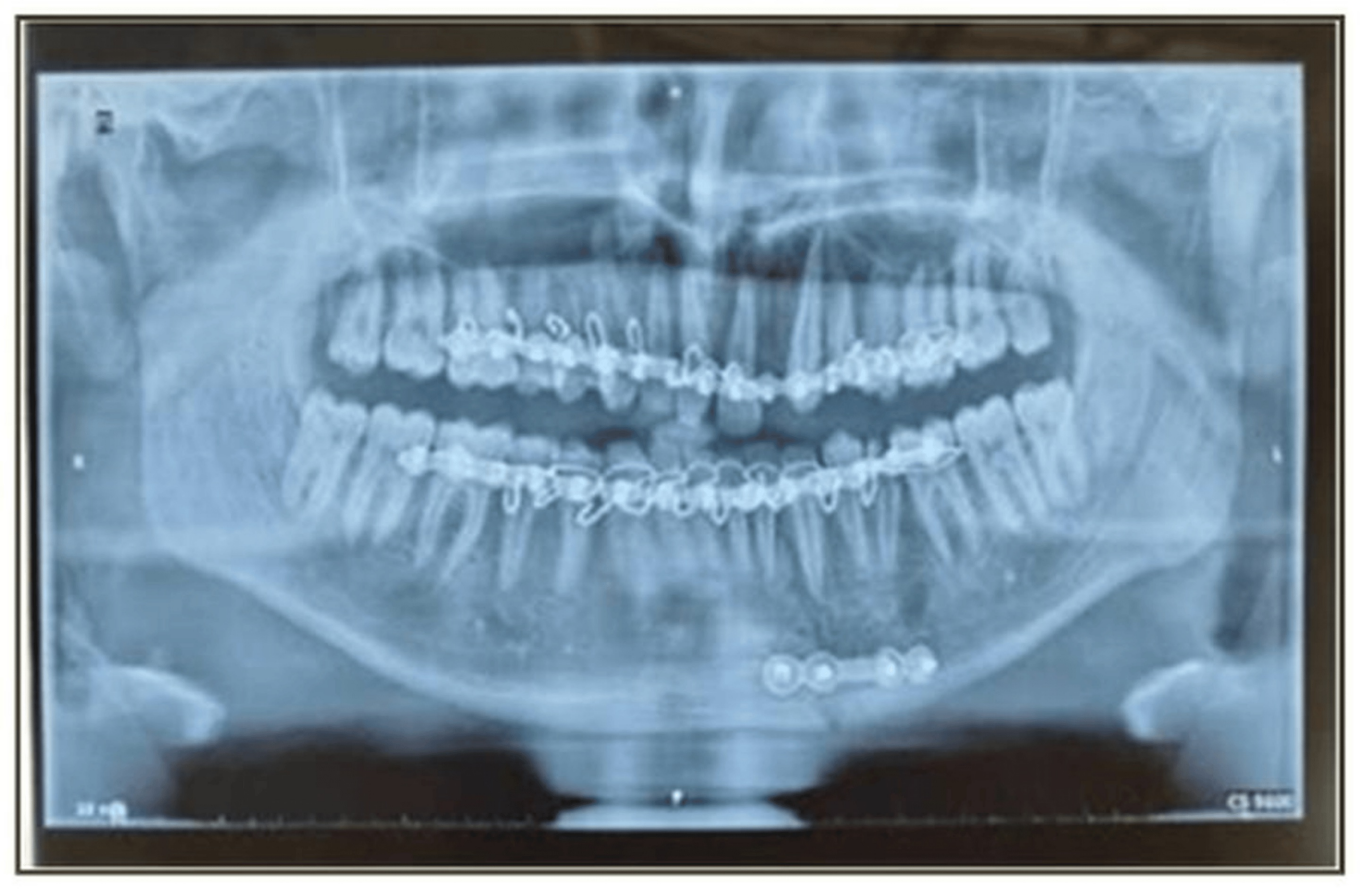
Postoperative orthopantomogram (OPG) at the end of four weeks

**Figure 9:**
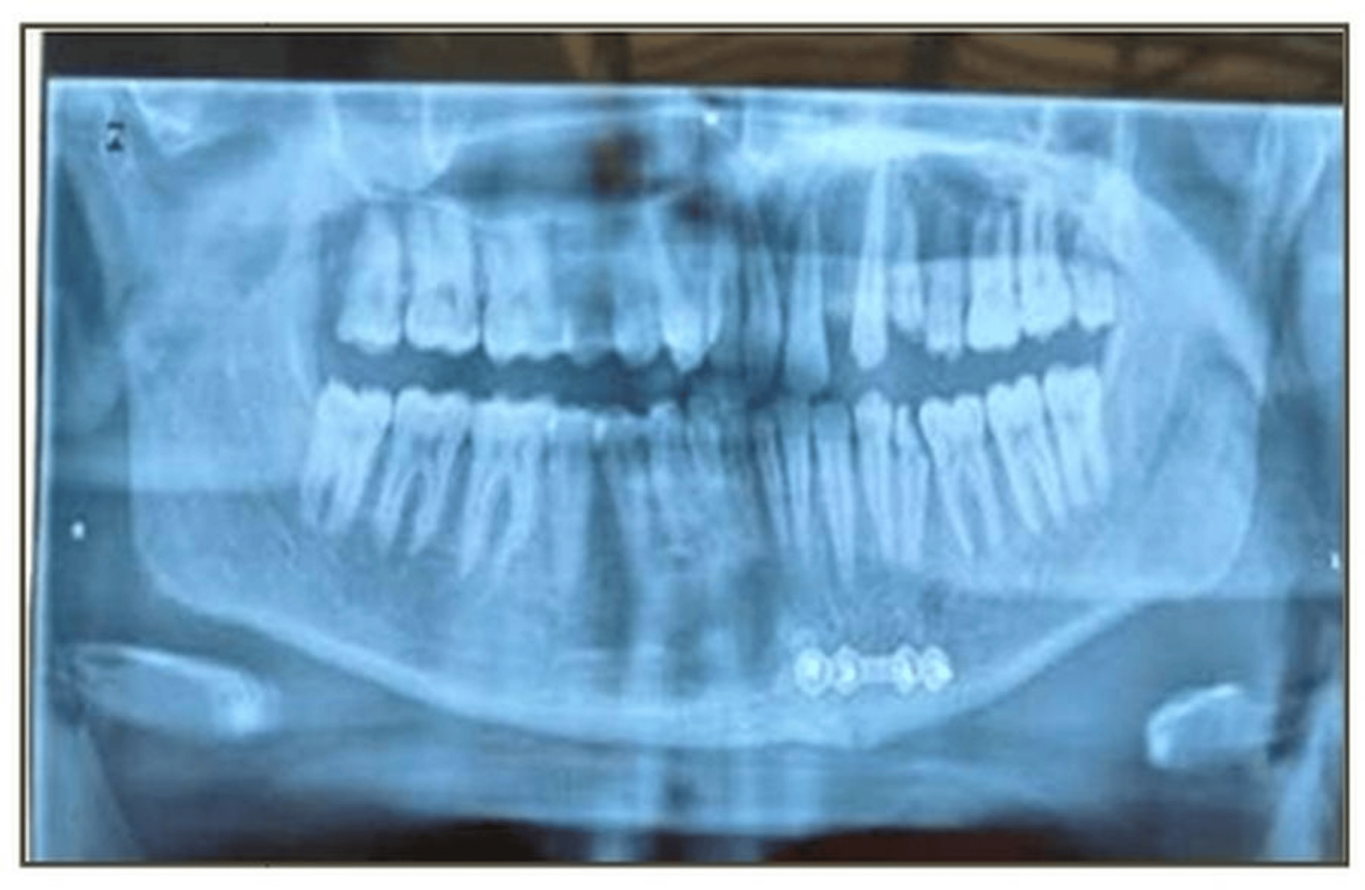
Postoperative orthopantomogram (OPG) at the end of 12 weeks

Postoperative assessments were conducted at one, four, and 12 weeks following surgery (Figures 6-9). During these intervals, several clinical parameters were systematically observed and recorded. Wound dehiscence was evaluated by noting the presence or absence of soft tissue separation at the surgical site. The incidence of bone or soft tissue infection was assessed clinically through signs such as inflammation, discharge, or radiographic changes. Malocclusion was examined by evaluating the patient’s dental alignment and occlusion. Plate exposure was monitored to determine whether any portion of the miniplate became visible through the mucosa or skin. Paresthesia in the mandibular or inferior alveolar nerve distribution was assessed by testing for sensory changes, including numbness or tingling in the lower lip and chin area. Plate fracture was identified either radiographically or clinically, based on signs of loosening or breakage of the miniplate. Finally, tenderness and mobility at the fracture site were checked to detect any ongoing instability or discomfort in the surgical region. Each of these parameters was evaluated at the designated follow-up intervals - week one, week four, and week 12 - and the findings were documented as either present (+) or absent (-).

## Results

Demographics

A total of 15 patients were included in the study, with a mean age of 35.73 ± 18.40 years (range: 19-78 years). Among them, 10 (66.7%) were male and five (33.3%) were female (Table 1).

**Table 1:** Demographics of the patients involved in the study (values are presented as number (percentage) or mean ± standard deviation)

Patient Data Summary	Value
Total Patients	15
Mean Age (± SD)	35.73 ± 18.40 years
Age Range	19 – 78 years
Male Patients	10 (66.7%)
Female Patients	5 (33.3%)

Etiology

The most common cause of mandibular fractures was road traffic accidents (RTAs), accounting for eight cases (53.4%), followed by falls (three cases, 20%), domestic abuse (two cases, 13.3%), and assault (two cases, 13.3%).

Fracture site and side

Fractures were distributed between the symphysis and parasymphysis regions. Parasymphysis fractures were slightly more common, observed in eight patients (53.4%), while symphysis fractures occurred in seven patients (46.6%). Regarding laterality, left-sided fractures were the most prevalent (seven cases, 46.6%), followed by right-sided fractures (five cases, 33.4%) and midline fractures (three cases, 20%).

Gender-based distribution

When stratified by gender, RTAs were the leading cause of fractures for both sexes, accounting for 60% of male cases (6/10) and 40% of female cases (2/5). Assault-related fractures were recorded only in males (2/10, 20%), whereas domestic abuse-related fractures were recorded only in females (2/5, 40%); falls occurred in both genders at equal proportions of 20% each (males 2/10, females 1/5). Regarding fracture site, symphysis fractures were proportionally more frequent in females (3/5, 60%) than in males (4/10, 40%), while parasymphysis fractures were more common in males (6/10, 60%) than in females (2/5, 40%). Side distribution showed that right-sided fractures were more common in males (4/10, 40%) than females (1/5, 20%), left-sided fractures were the most frequent overall (males 5/10, 50%; females 2/5, 40%), and midline fractures occurred more often in females (2/5, 40%) than in males (1/10, 10%).

Postoperative complications

Complications were minimal during the follow-up period. No cases of plate exposure, wound dehiscence, plate fracture, fracture non-union/malunion, or mobility at the fracture site were observed (Table 2).

**Table 2:** Postoperative complications observed in the 15 patients involved in the study

Complication	N	%
Plate exposure	0	0%
Nerve paresthesia	1	6.70%
Malocclusion	1	6.70%
Wound dehiscence	0	0%
Bone or soft tissue infection	1	6.70%
Plate fracture	0	0%
Tenderness and mobility at fracture site	0	0%
Fracture non-union/malunion	0	0%

However, nerve paresthesia was reported in one patient (6.7%), consistent with rates of inferior alveolar nerve disturbance reported in mandibular trauma literature [1]. Malocclusion was observed in one patient (6.7%), which resolved without further surgical intervention. Infection involving the soft tissue occurred in one patient (6.7%), which was managed conservatively with antibiotics.

Three complications were noted, each in one patient (6.7%): nerve paresthesia, malocclusion, and soft tissue infection. No cases of plate exposure, wound dehiscence, plate fracture, fracture non-union/malunion, or mobility at the fracture site were reported. These results are in line with earlier studies on postoperative complication rates in mandibular fracture management [1].

## Discussion

Fractures of the mandibular symphysis and parasymphysis constitute approximately 18-20% of mandibular fractures in adults and are second only to fractures of the angle and condyle in terms of frequency [11]. Their prevalence is influenced by anatomical weaknesses such as the presence of the mental foramen, deep-rooted canine sockets, and the curvature of the mandibular arch, which render these areas more susceptible to fracture during trauma [12]. The majority of such injuries, as corroborated by our study, result from road traffic accidents, which remain a leading cause of maxillofacial trauma in developing countries [13,14].

Biomechanically, the anterior mandible experiences complex overlapping forces - tensile, compressive, and torsional - unlike the more predictable stress distributions found in the mandibular body and angle [15]. The action of masticatory muscles, including the masseter, temporalis, and medial pterygoid, contributes to the torsional forces across the symphysis and parasymphysis. These forces are further intensified when occlusal loading is involved, especially in unilateral or parasymphyseal fractures [16]. This complexity makes achieving stable fixation in this region particularly challenging.

Historically, two philosophies have governed the management of mandibular fractures: the AO/ASIF system, which advocates for rigid fixation with load-bearing plates, and Champy's technique, which promotes functional stability using monocortical miniplates placed along osteosynthesis lines [6,17]. Champy's concept is particularly relevant in anterior mandibular fractures, where stress is concentrated along the superior border. In such cases, a sub-apical plate is recommended to neutralize tensile forces, while an inferior border plate resists compressive forces.

However, studies have raised concerns about the complications associated with dual plating in the parasymphysis. The subapical region, located near the apices of the canine and premolar teeth and close to the mental foramen, presents limited space for screw placement and increases the risk of root injury and neurovascular compromise [18]. Our findings echo these concerns, as no major infections or plate exposures occurred, supporting previous studies that suggest increased complication rates with two-plate systems [18,19].

The use of a single miniplate at the lower border, in conjunction with an arch bar acting as a tension band, offers several advantages. It simplifies the surgical procedure, reduces operative time, minimizes hardware-related complications, and maintains occlusal stability. Several studies have confirmed that tension band principles can be effectively achieved using arch bars alone in select cases, obviating the need for a second plate [20,21]. This method also reduces the risk of iatrogenic injury to dental roots and the mental nerve. However, this study's limited sample size underscores the need for further research with larger cohorts and long-term follow-up to assess its applicability in more complex mandibular fracture patterns, including comminuted and panfacial injuries.

In our study, one patient experienced temporary mental nerve paresthesia, which resolved within eight weeks. This transient neuropathy likely resulted from intraoperative retraction or manipulation, rather than direct nerve injury, and is a known risk with intraoral access to the inferior mandibular border [22]. The frequency and duration of paresthesia in our series were comparable to other reports in the literature.

No cases of iatrogenic root injury were noted on postoperative imaging. However, several anatomical studies have shown that the buccal cortical bone in the premolar region is often less than 2.5 mm thick, and the proximity of roots makes the area high-risk for screw penetration, especially in female patients with narrower mandibles [23]. Thus, proper preoperative imaging and cautious plate positioning are essential for avoiding dental damage.

Additionally, none of our patients required plate removal, which may be attributed to careful patient selection, oral hygiene maintenance, and the avoidance of subapical plating in patients with reduced mandibular height. The literature consistently shows that plate removal is more frequently required in cases involving dual plating due to exposure, loosening, or infection [22].

Overall, our findings affirm that single lower border miniplate fixation, supported by a tension band mechanism via an arch bar, is a reliable and biomechanically sound treatment for mandibular parasymphysis fractures. This method provides sufficient stability for bone healing, minimizes complications, and respects the regional anatomy. However, this study's limited sample size underscores the need for further research with larger cohorts and long-term follow-up to assess its applicability in more complex mandibular fracture patterns, including comminuted and panfacial injuries.

## Conclusions

This prospective study evaluated the efficacy of using a single miniplate for the fixation of mandibular symphysis and parasymphysis fractures in a sample of 15 patients. The results demonstrated that this approach yields favorable clinical outcomes with minimal complications. No cases of fracture nonunion, malunion, or screw-related failure were observed. A single instance of minor wound dehiscence and one case of transient mental nerve paresthesia were the only complications noted, both of which resolved without long-term consequences.

The use of a single miniplate along the inferior border, combined with an Ehrich’s arch bar as a tension band, effectively counteracted tensile forces and ensured functionally stable fixation. Importantly, no iatrogenic dental root or neurovascular injuries were observed, reinforcing the safety of this approach in anatomically constrained regions of the mandible. Given its lower cost and reduced risk profile, single miniplate fixation appears to be a reliable and economically viable alternative to dual-plate fixation, especially in resource-constrained settings like India. 

## References

[1] Ellis E 3rd, Moos KF, el-Attar A (1985). Ten years of mandibular fractures: an analysis of 2,137 cases. Oral Surg Oral Med Oral Pathol.

[2] Lee KH (2008). Epidemiology of mandibular fractures in a tertiary trauma centre. Emerg Med J.

[3] Tiwana PS, Kushner GM, Alpert B (2007). Lag screw fixation of anterior mandibular fractures: a retrospective analysis of intraoperative and postoperative complications. J Oral Maxillofac Surg.

[4] Subhashraj K, Nandakumar N, Ravindran C (2007). Review of maxillofacial injuries in Chennai, India: a study of 2748 cases. Br J Oral Maxillofac Surg.

[5] Fonseca RJ, Walker RV (2013). Oral and Maxillofacial Trauma. 4th ed.

[6] Champy M, Lodde JP, Schmitt R, Jaeger JH, Muster D (1978). Mandibular osteosynthesis by miniature screwed plates via a buccal approach. J Maxillofac Surg.

[7] Wusiman P, Abasi K, Maimaitishawuti D, Moming A (2019). Management of mandibular angle fractures using one miniplate or two miniplate fixation system: a systematic review and meta-analysis. J Oral Maxillofac Surg.

[8] Ellis E, Ghali GE (1991). Lag screw fixation of symphysis and parasymphysis fractures of the mandible. J Oral Maxillofac Surg.

[9] Natu SS, Pradhan H, Gupta H (2012). An epidemiological study on pattern and incidence of mandibular fractures. Plast Surg Int.

[10] Naing L, Nordin RB, Abdul Rahman H, Naing YT (2022). Sample size calculation for prevalence studies using Scalex and ScalaR calculators. BMC Med Res Methodol.

[11] Subhashraj K, Ramkumar S, Ravindran C (2008). Pattern of mandibular fractures in Chennai, India. Br J Oral Maxillofac Surg.

[12] Gassner R, Tuli T, Hächl O, Moreira R, Ulmer H (2003). Craniomaxillofacial trauma: a 10-year review of 9,543 cases with 21,067 injuries. J Oral Maxillofac Surg.

[13] Boffano P, Roccia F, Zavattero E (2015). European Maxillofacial Trauma (EURMAT) project: a multicentre and prospective study. J Craniomaxillofac Surg.

[14] Chrcanovic BR (2013). Open versus closed reduction: comminuted mandibular fractures. Oral Maxillofac Surg.

[15] Haug RH, Prather J, Indresano AT (1990). An epidemiologic survey of facial fractures and concomitant injuries. J Oral Maxillofac Surg.

[16] Zide MF, Kent JN (1983). Indications for open reduction of mandibular fractures. J Oral Maxillofac Surg.

[17] Rai A, Datarkar A, Borle RM (2011). Are maxillomandibular fixation screws a better option than Erich arch bars in achieving maxillomandibular fixation? A randomized clinical study. J Oral Maxillofac Surg.

[18] Chrcanovic BR, Freire-Maia B, Souza LN, Araújo VO, Abreu MH (2004). Facial fractures: a 1-year retrospective study in a hospital in Belo Horizonte. Braz Oral Res.

[19] Erol B, Tanrikulu R, Görgün B (2004). Maxillofacial fractures. Analysis of demographic distribution and treatment in 2901 patients (25-year experience). J Craniomaxillofac Surg.

[20] Ellis E III, Walker L (1996). Treatment of mandibular angle fractures using one noncompression miniplate. J Oral Maxillofac Surg.

[21] Goodday RH (2013). Management of fractures of the mandibular body and symphysis. Oral Maxillofac Surg Clin North Am.

[22] Pogrel MA, Kaban LB (1993). Injuries to the inferior alveolar and lingual nerves. J Calif Dent Assoc.

[23] Lee HE, Han SJ (2018). Anatomical position of the mandibular canal in relation to the buccal cortical bone: relevance to sagittal split osteotomy. J Korean Assoc Oral Maxillofac Surg.

